# Positive End-Expiratory Pressure Setting in COVID-19-Related Acute Respiratory Distress Syndrome: Comparison Between Electrical Impedance Tomography, PEEP/FiO_2_ Tables, and Transpulmonary Pressure

**DOI:** 10.3389/fmed.2021.720920

**Published:** 2021-12-22

**Authors:** Sébastien Gibot, Marie Conrad, Guilhem Courte, Aurélie Cravoisy

**Affiliations:** Service de Réanimation Médicale, Hôpital Central, CHRU, Nancy, France

**Keywords:** ARDS, COVID-19, PEEP, electrical tomography impedance, mechanical ventilation

## Abstract

**Introduction:** The best way to titrate the positive end-expiratory pressure (PEEP) in patients suffering from acute respiratory distress syndrome is still matter of debate. Electrical impedance tomography (EIT) is a non-invasive technique that could guide PEEP setting based on an optimized ventilation homogeneity.

**Methods:** For this study, we enrolled the patients with 2019 coronavirus disease (COVID-19)-related acute respiratory distress syndrome (ARDS), who required mechanical ventilation and were admitted to the ICU in March 2021. Patients were monitored by an esophageal catheter and a 32-electrode EIT device. Within 48 h after the start of mechanical ventilation, different levels of PEEP were applied based upon PEEP/FiO_2_ tables, positive end-expiratory transpulmonary (P_L_)/ FiO2 table, and EIT. Respiratory mechanics variables were recorded.

**Results:** Seventeen patients were enrolled. PEEP values derived from EIT (PEEP_EIT_) were different from those based upon other techniques and has poor in-between agreement. The PEEP_EIT_ was associated with lower plateau pressure, mechanical power, transpulmonary pressures, and with a higher static compliance (Crs) and homogeneity of ventilation.

**Conclusion:** Personalized PEEP setting derived from EIT may help to achieve a more homogenous distribution of ventilation. Whether this approach may translate in outcome improvement remains to be investigated.

## Introduction

Despite progresses in acute respiratory distress syndrome (ARDS) management, the best way to titrate a positive end-expiratory pressure (PEEP) is not straightforward (1). The “right” PEEP should allow for optimized lung recruitment while minimizing over-distention. To this aim, clinicians can use PEEP-FiO_2_ tables (2), transpulmonary pressure (P_L_) (3), or electrical impedance tomography (EIT).

The transpulmonary pressure is measured using an esophageal balloon catheter that approximates the pleural pressure. Using this technique, PEEP has to be set to maintain the end-expiratory P_L_ above zero to avoid collapse of dependent dorsal lung regions, and the end-inspiratory P_L_ below 20–25 cmH_2_O to decrease the risk of overdistension of non-dependent regions.

Electrical impedance tomography (EIT) is a non-invasive technique giving dynamic information on regional ventilation that can be embarked in modern ventilators. Regional hypoventilated lung units (“Silent spaces”) correspond to both collapsed areas in the dependent territories, and distended areas in the non-dependent regions. Using this technique, PEEP is set to minimize the percentage of total silent spaces.

We describe a case series of patients suffering from 2019 coronavirus disease (COVID-19)-related ARDS in whom we compared PEEP settings based on PEEP/FiO_2_ tables, P_L_/ FiO2 table, and EIT.

## Methods

In March 2021, we enrolled some mechanically ventilated patients who were admitted to our Intensive Care Unit (ICU) because of a COVID-19-related moderate-to-severe ARDS. The diagnosis of COVID-19 relied upon positive result on polymerase chain reaction of sputum or nasal swab. The Ethic Committee of our University Hospital approved this study with a waiver of informed consent because of the use of routine procedures, as well as the use of de-identified data.

All patients were ventilated in volume control mode [tidal volume (Vt): 6–7 ml/kg ideal body weight (IBW)], with FiO_2_ set to achieve peripheral oxygen saturation (SpO_2_) between 92 and 95%, and respiratory rate (RR) set to reach PaCO_2_ between 38 and 45 mmHg. Transpulmonary pressures were measured with the use of an esophageal balloon catheter (Nutrivent; Sidam, Mirandola, Italy) after its correct positioning has been verified through passive chest compression during occlusion. As part of our routine monitoring, patients were also equipped with a 32-electrode soft-textile EIT belt (Sentec; Therwil, Switzerland), which was directly connected to the ventilator (ELISA 800 VIT, Lowenstein Medical; Kronberg, Germany). Some maneuvers were performed in supine position after 24–48 h of mechanical ventilation while the patients were still sedated (midazolam and sufentanyl) and paralyzed (cisatracurium or atracurium). Respiratory mechanics variables were recorded after 10 min at different PEEP levels while all the other parameters (FiO_2_, Vt, RR, flow rates, etc.) remained unchanged.

Positive end-expiratory pressure (PEEP) was first set according to the lower, then, to the higher PEEP/FiO_2_ ALVEOLI table (2). Next, PEEP as based upon end-expiratory P_L_/FiO_2_ table, was applied (3). Finally, an automated decremental PEEP trial was performed under EIT monitoring (Best-PEEP-Tool, Lowenstein Medical): PEEP was set at 24 cmH_2_O (corresponding to the maximum PEEP in the PEEP/FiO_2_ table) and was reduced by 2 cmH_2_O every 10 inspirations until 6 cm H_2_O, with a 3-s end-expiratory hold between decremental steps. For each PEEP values, percentages of relatively collapsed and overdistended lung regions were given by the EIT, and the “best” PEEP (PEEP_EIT_) was considered as the lowest level associated with the lowest total percentage of the lung silent spaces (collapsed + distended).

Data are presented as median (interquartile range) and are compared using Wilcoxon signed-rank test. Bias and limits of agreement between different approaches were calculated with the Bland-Altman approach. Statistical analyses were performed by GraphPad software (La Jolla, CA, USA) with two-tailed *p* < 0.05 deemed as significant.

## Results

Seventeen patients (15 men, 2 women) were enrolled. Median age was 65 (62–71) years, and body mass index was 31.1 (28.5–33.0). The ARDS was severe in 6 and moderate in 11 patients, while the PaO_2_/FiO_2_ was 136 (103–155), Vt 6.6 (6.2–7.0) mL/kg IBW, and RR 24 (22–27), respectively. Twelve patients were under high-flow oxygen therapy for a median of 1 (1, 2) day before intubation. All patients, except for one, were discharged alive.

**Figure 1:**
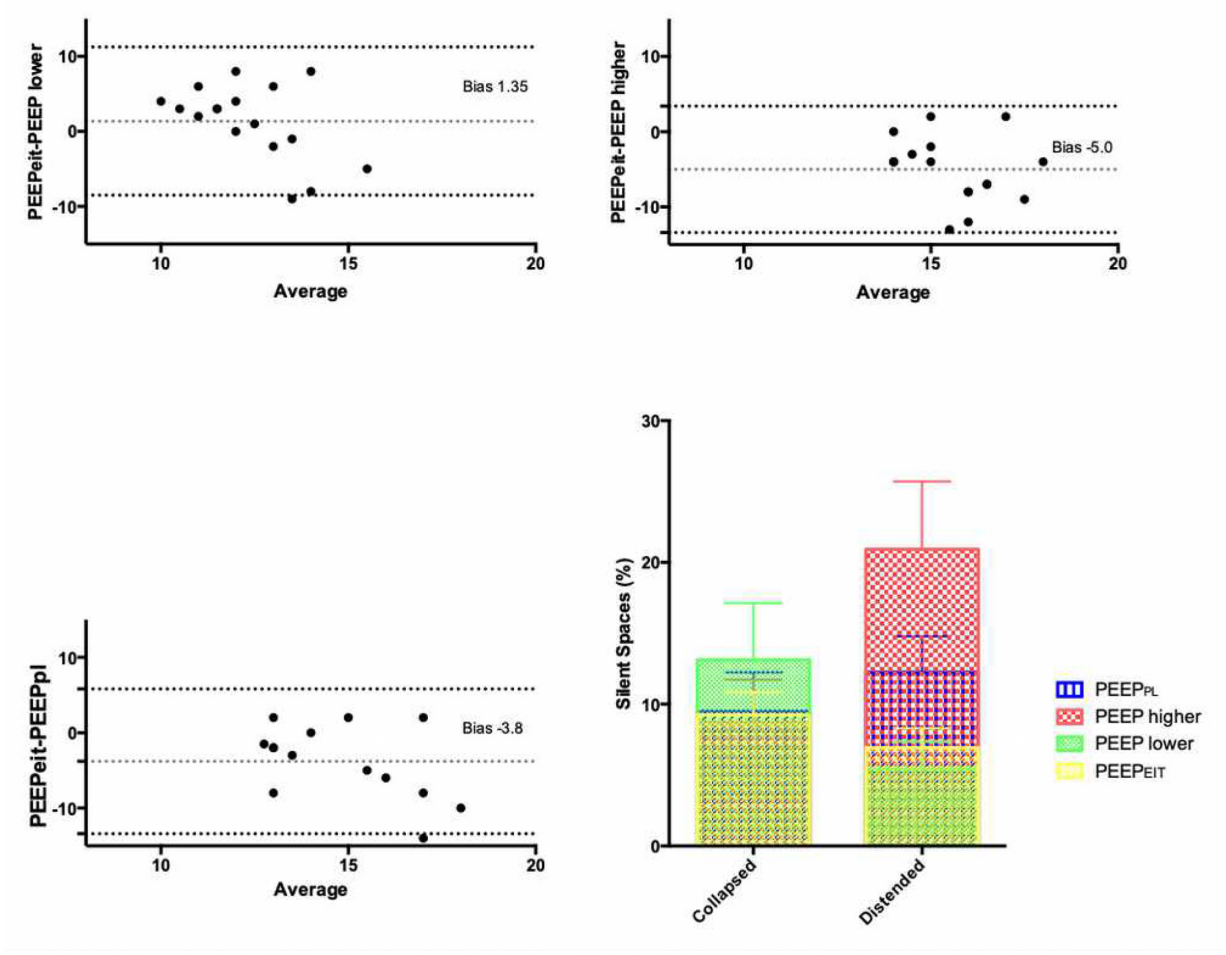
Bland-Altman plots evaluating agreement between PEEP derived from EIT (PEEP_EIT_) and positive end-expiratory pressure (PEEP) values derived from lower and higher PEEP/FiO_2_ tables and P_L_/FiO_2_ table. Dotted lines: bias and its 95% confidence interval. Lower right panel: percentages of collapsed and distended lung regions measured by electrical impedance tomography (EIT) under different PEEP settings. Percentage of collapse was lower with PEEP_EIT_ than with lower PEEP/FiO_2_ table (*p* = 0.04), while percentage of distended areas was reduced as compared to higher PEEP/FiO_2_ and P_L_/FiO_2_ tables (*p* < 0.01).

Positive end-expiratory pressure derived from EIT (PEEP_EIT_), corresponding to the lowest level of PEEP achieving the lowest percentage of total silent spaces (distended + collapsed), was significantly different from the other PEEP values. It was higher than the lower PEEP/FiO_2_ table, and lower than the higher PEEP/FiO_2_ or P_L_/FiO_2_ tables (Table 1). The Bland-Altman analysis showed that PEEP_EIT_ was 1.3 cm H_2_O higher than the lower PEEP/FiO_2_ table with limits of agreement from −8.5 to 11.2 cm H_2_O. By contrast, PEEP_EIT_ was 5 and 4 cm H_2_O lower, respectively, than higher PEEP/FiO_2_ and P_L_/FiO_2_ tables, with again wide limits of agreement (Figure 1).

**Table 1:** Effect of positive end-expiratory pressure (PEEP) settings on respiratory mechanics.

**Variable**	**Lower PaO_2_/FiO_2_ table**	**Higher PaO_2_/FiO_2_ table**	**P_L_/FiO_2_ table**	**PEEP_EIT_**
PEEP (cm H_2_O)	10 (10 to 14)	17 (16 to 20)	15 (14 to 20)	13 (12 to 14)^*^^#^
P_PLAT_ (cm H_2_O)	23 (20 to 26)	33 (28 to 38)	29 (24 to 37)	25 (22 to 27)^#^
Driving Pressure (cm H_2_O)	12 (10 to 14)	14 (12 to 18)	13 (11 to 16)	12 (11 to 13)
C_RS_ (mL/cm H_2_O)	39 (34 to 48)	30 (24 to 37)	35 (26 to 43)	38 (34 to 45)^#^
Mechanical Power (J/min)	25.1 (22.7 to 34.3)	34.4 (28.0 to 43.6)	34.1 (26.0 to 40.7)	28.4 (24.4 to 32.0)^#^
Inspiratory Transpulmonary pressure (cm H_2_O)	6.6 (4.3 to 13.5)	17.5 (9.9 to 21.6)	14.7 (9.4 to 18.7)	11.5 (6.4 to 14.3)^#^
Expiratory Transpulmonary pressure (cm H_2_O)	−0.3 (−2.8 to 3.6)	5.0 (2.0 to 8.0)	4.0 (1.5 to 6.5)	1.3 (0.1 to 2.0)^#^
Silent spaces (%)	18 (10 to 26)	30 (13 to 48)	23 (17 to 35)	16 (9 to 23)^#^

* *p < 0.05 PEEP_EIT_ vs. Lower PaO_2_/FiO_2_ table.*

# *p < 0.05 PEEP_EIT_ vs. Higher PaO_2_/FiO_2_ and P_L_/FiO_2_ tables*.

In terms of respiratory mechanics, PEEP_EIT_ was associated with lower plateau pressure, mechanical power, transpulmonary pressures, and with a higher static compliance (Crs) than higher PEEP/FiO_2_ or P_L_/FiO_2_ tables (Table 1). Driving pressures were not significantly different.

A better distribution of ventilation was achieved with PEEP_EIT_: lung collapse was lower with PEEP_EIT_ than with lower PEEP/FiO_2_ table (9 vs. 13%; *p* = 0.04), while lung distension was reduced as compared to higher PEEP/FiO_2_ and P_L_/FiO_2_ tables (6 vs. 20 and 13%, respectively; *p* < 0.01) (Figure 1).

## Discussion

Personalized PEEP guided by EIT, with the aim to minimize relative alveolar distention and collapse, was different than PEEP based upon PEEP/FiO_2_ or P_L_/FiO_2_ tables. Although in terms of respiratory mechanics, PEEP_EIT_ did not differ from lower PEEP/FiO_2_ table. There were very important individual variations as witnessed by the wide range of limit agreement in Bland-Altman analyses. Therefore, each patient exhibited different lung properties that cannot be ascertained by using global mechanics parameter such as driving or transpulmonary pressures, compliance, or pressure-volume curves. This may explain the negative results of important clinical trials, which compared low vs. high PEEP in ARDS patients (2, 4).

Several other recent studies evaluated EIT-guided PEEP titration. Van der Zee et al. (5) and Sella et al. (6) have compared PEEP_EIT_ vs. PEEP/FiO_2_ tables in each of the 15 cases of COVID-19-related ARDS patients. In both studies, PEEP values differed with important individual variations. Interestingly, PEEP_EIT_ was lower (12 cmH_2_O) in the Sella study than in the Van der Zee's (21 cm H_2_O). This highlights the huge variability between patients with ARDS, depending on weight, age, sex, or duration of mechanical ventilation.

When comparing PEEP_EIT_ and P_L_/FiO_2_ table, Scaramuzzo et al. (7), in 20 patients under non-COVID-19 ARDS, found no correlation between the values given by the 2 techniques. As in our study, PEEP_EIT_ achieved a more homogenous distribution of ventilation.

Our work has several limitations. First, we only included patients with COVID-19. Whether these patients behaved similarly to those suffering from non-COVID-19 ARDS in terms of respiratory mechanics is still matter of debate. Second, most of our patients were over-weighted. Hence, this may have contributed to the low agreement between techniques. Finally, only 17 patients have been enrolled, precluding any generalization. However, each patient was its own control, and we just wanted to underline the poor agreement between routinely used techniques at the patient level.

The use of EIT allows for a personalized PEEP titration with the aim to minimize the total amount of pulmonary silent spaces. Whether this approach could translate in outcome improvement remains to be investigated.

## Data Availability Statement

The raw data supporting the conclusions of this article will be made available by the authors, without undue reservation.

## Ethics Statement

The studies involving human participants were reviewed and approved by Comité d'éthique du CHRU de Nancy. Written informed consent for participation was not required for this study in accordance with the national legislation and the institutional requirements.

## Author Contributions

SG designed the study, collected and analyzed data, and wrote the manuscript. MC, GC, and AC collected and analyzed data. All authors read and approved the manuscript.

## Conflict of Interest

The authors declare that the research was conducted in the absence of any commercial or financial relationships that could be construed as a potential conflict of interest.

## Publisher's Note

All claims expressed in this article are solely those of the authors and do not necessarily represent those of their affiliated organizations, or those of the publisher, the editors and the reviewers. Any product that may be evaluated in this article, or claim that may be made by its manufacturer, is not guaranteed or endorsed by the publisher.
